# Information architectures: a framework for understanding socio-technical systems

**DOI:** 10.1038/s44260-025-00037-z

**Published:** 2025-04-17

**Authors:** Paul E. Smaldino, Adam Russell, Matthew R. Zefferman, Judith Donath, Jacob G. Foster, Douglas Guilbeault, Martin Hilbert, Elizabeth A. Hobson, Kristina Lerman, Helena Miton, Cody Moser, Jana Lasser, Sonja Schmer-Galunder, Jacob N. Shapiro, Qiankun Zhong, Dan Patt

**Affiliations:** 1 Department of Cognitive and Information Sciences, University of California-Merced, Merced, CA USA; 2 Santa Fe Institute, Santa Fe, NM USA; 3 Information Sciences Institute, University of Southern California, Los Angeles, CA USA; 4 Department of Defense Analysis, Naval Postgraduate School, Monterey, CA USA; 5 Berkman Klein Center, Harvard University, Cambridge, MA USA; 6 Departments of Informatics and Cognitive Science, Indiana University-Bloomington, Bloomington, IN USA; 7 Graduate School of Business, Stanford University, Stanford, CA USA; 8 Department of Communication, University of California-Davis, Davis, CA USA; 9 Department of Biological Sciences, University of Cincinnati, Cincinnati, OH USA; 10 IDea_Lab, University of Graz, Graz, Austria; 11 Complexity Science Hub Vienna, Vienna, Austria; 12 Department of Computer & Information Science & Engineering, University of Florida, Gainesville, FL USA; 13 Department of Politics, School of Public and International Affairs, Princeton University, Princeton, NJ USA; 14 Max Planck Institute for Human Development, Berlin, Germany; 15 Hudson Institute, Washington, DC USA

**Keywords:** Social sciences, Technology

## Abstract

A sequence of technological inventions over several centuries has dramatically lowered the cost of producing and distributing information. Because societies ride on a substrate of information, these changes have profoundly impacted how we live, work, and interact. This paper explores the nature of *information architectures* (IAs)—the features that govern how information flows within human populations. IAs include physical and digital infrastructures, norms and institutions, and algorithmic technologies for filtering, producing, and disseminating information. IAs can reinforce societal biases and lead to prosocial outcomes as well as social ills. IAs have culturally evolved rapidly with human usage, creating new affordances and new problems for the dynamics of social interaction. We explore societal outcomes instigated by shifts in IAs and call for an enhanced understanding of the social implications of increasing IA complexity, the nature of competition among IAs, and the creation of mechanisms for the beneficial use of IAs.

## Introduction

“We are but whirlpools in a river of ever-flowing water. We are not stuff that abides, but patterns that perpetuate themselves. A pattern is a message, and may be transmitted as a message”.

–Norbert Wiener^1^ *The Human Use of Human Beings: Cybernetics and Society*

Technology, human society, and communication networks have always been intertwined—from the earliest footpath networks that enabled trade between small groups of people to today’s globalized economy connected by Internet-enabled smartphones. However, the tremendous growth in the scale and capabilities for information exchange has yielded something new that is not yet fully understood. As Philip Anderson^2^ famously observed of physical systems, “more is different.” The same is often true of social systems. Building on the sciences of the early 19th century—physics, chemistry, thermodynamics, electromagnetics—humanity built amazing inventions, creating the lightbulb, telephone, automobile, and airplane in a 30-year span. But it wasn’t these inventions alone that drove economic growth and transformed society; it was also the networks they spawned—an electrical grid, a telephone network, a highway system, and an air transportation system. With the growth of information technologies, these networks became enmeshed into the fabric of our social and cultural organizations. Most recently, AI algorithms, including large language models (LLMs), have become rapidly adopted across many industries and threaten further disruption of the social fabric through their influence on information flow^3^.

Over the past century, the costs of creating, distributing, and processing information have dramatically decreased. These extreme discounts have disrupted the economics of socio-technical systems, stripping distributors like newspapers, telephone companies, or TV networks of power and fomenting new competitions for human attention. In just the last century, we have gone from a small number of media outlets competing for overlapping market share to a large number of outlets exploiting niche audiences to competing repositories where anyone can generate and disseminate content^4,5^. While an individual in a developed country can refrain from using certain technologies—e.g., abstaining from smartphone use or subscription payments—doing so increasingly means effectively withdrawing from society. Engaging in trade, obtaining housing or education, and even finding romantic partners all depend on some substrate of underlying technical and information networks. The control over the flow of information—which now includes not only direct communication but nearly all modern exchanges of financial and social capital—is one of the pivotal levers involved in the power struggles of contemporary geopolitics^6^.

Technologies are, for the most part, neither good nor bad by themselves, but they do change how people can organize, communicate with, and even exploit one another. As McLuhan remarked, “We shape our tools, and thereafter, they shape us”^7^. On one side, there are the considerable economic gains that have come with technology. There is much less global poverty, childhood death, and arguably global suffering than there was two centuries ago. On the other hand, technologies have also exacerbated a slew of social ills, including political polarization^8^, socioeconomic inequality^9^, mental health crises^10^, and societal discord^5^. We are also beginning to recognize that architectures can vary both within and between states, with implications for the future of choice with respect to values and ethics.

If human societies and information technologies are inexorably coupled, then it is imperative to understand the dynamics involved in those couplings, and how the specific features of the information technologies that tie a group of people together shape and are shaped by those same people. We use the term *information architecture* (IA) to concisely describe this concept. There is already a great deal of research into techno-social systems, with burgeoning studies of social networks, mesoeconomics, and cultural evolution^3,11–15^. However, there are also many gaps in this work, and few efforts to understand how different information architectures interact, cooperate, and compete. We offer the beginnings of a unifying, interdisciplinary framework to help us build a desperately needed social science for the 21st century.

## What are information architectures?

The term “information architecture” is not new, and dates back to at least the 1970s; it has been used within some communities of organizational science, urban design, software development, and user experience (UX) research^16–20^. In these contexts, information architecture typically refers to a process of intentional, active design as performed by one or more “information architects.” A representative definition of information architecture in this literature is the “organization of information to support findability, manageability, and usefulness from the infrastructural level to the user interface level”^18^. The information architect’s job, in this context, is to design systems with users in mind, to facilitate the best possible user experience and the highest-value engagement with information in those systems^20^.

We aim to extend the traditional conception of IAs from this literature in two ways: first, to expand it beyond the scale of specific organizations, applications, or interfaces to encompass the flow of information throughout society, and second, to expand the concept of “design” to include non-deliberate processes. We are interested in information architectures that result from the combination of both deliberate and non-deliberate processes that unfold over multiple time scales. Although our perspective on information architectures differs from past usage, we adopt the term because, at heart, we still seek to describe and understand how the nature of social and technological organization facilitates how information is or is not accessed and transmitted.

For the remainder of this paper, we define information architecture as *the set of structural features and affordances that enables and constrains information flow between sources and receivers within a population*. By structural features, we include both physical infrastructure—such as transportation networks, broadcast media, telecom and cell networks, geographical features (e.g., mountains dividing populations, rivers connecting them)—and cultural features, including social norms, laws, languages, and both formal and informal institutions. We also include digital infrastructures and other mechanisms for online communication via computers or smartphones, including peer-to-peer communication channels (e.g., SMS, WhatsApp, WeChat), social media platforms (e.g., Facebook, Instagram, Telegram, Twitter/X, TikTok), blogs and related platforms (e.g., Medium, Substack), and other conduits for assessing or storing information (e.g., blockchain, banks, public records, etc.). Such features can enable information flow (e.g., by allowing individuals to send messages or access stored information) and constrain it (e.g., by restricting certain types of content or the set of individuals who can send or receive messages or access certain information). By affordances, we mean the opportunities and behaviors made possible by those structural features^21,22^, as well as the means of controlling those features.

Given the extent to which our modern world is connected, there is, in some sense, only one all-encompassing information architecture, encompassing every individual on the planet. However, this perspective ignores the extent to which subpopulations can be functionally defined based on national, linguistic, ethnic, and other boundaries. Different groups and subgroups can experience different information architectures. A focus on IAs may thus provide insight into the diversification and maintenance of subpopulations. For example, as groups develop different IAs, their organizational capabilities change, and they increasingly undergo different evolutionary trajectories.

There is a large literature on the relationship between the structure of social networks and the diffusion of information on those networks^14,23–27^. Indeed, information architectures include conduits for diffusion, but the characterization of an information architecture goes beyond merely what is being diffused and the extent of that diffusion. Such a characterization also includes consideration of the identity of individuals transmitting or receiving information, the accessibility of information, and how the packaging of information can change its nature. A key aspect of our treatment of information architectures is how they interact with one another, a feature usually omitted from discussions of diffusion. Even when diffusion studies consider multiple groups, they rarely highlight differences in how information flows both within and between groups (for some exceptions, see refs. ^28–30^). Information architectures can, therefore, be characterized along several dimensions, and these means of characterization are important for comparing and contrasting distinct architectures and considering how they might compete.

## Characterizing information architectures

As an illustrative model, consider two fictitious societies. These are caricatures but represent two extreme poles of information control in the industrialized world, which will help us explore the consequences of the institutions that shape the information architectures within each of them.

The first of these is called *Harmonia*. Its institutions are intended to produce an absence of discord in the interest of social stability—carefully filtering outside information and regulating internal interactions, curation algorithms, and content to weed out misinformation or framings that might sow discontent. Because of the vast complexity of these tasks and the difficulty of employing consistent means of decision-making, control is centralized with the leadership of Harmonia. Naturally, this leadership will wield its power over the content people can consume with great care, while the interests of the leadership and the people—in terms of concerns for accuracy, well-being, and freedom—will not always align.

The second of these societies is called *Libertaria*. Its institutions value individual choice above all, resisting interventions or regulations by leaders concerning information flows. Because any imposition of control has the potential for abuse and quashing of minority voices, no top-down control is granted to any central authority, though inequality of wealth inevitably leads to inequality of influence. The people in Libertaria are thus immersed in a sea of conflicting information, with topics often driven by attention-maximizing algorithms, but they are also free to speak their minds and to seek out those whose beliefs and goals align with their own, even when these are at odds with societal harmony.

The study of information architectures concerns itself with understanding the relative benefits and costs of living in a particular society, the interactions between societies, the interventions that citizens and leaders might engage in, and ways in which societies transition between alternative structures to address functional needs or respond to shocks. In this sense, IAs can be viewed as group-level traits possessed by a society, though they are also part of the larger environmental niche in which that society’s inhabitants exist^31^. It might be that certain kinds of IAs enable greater flexibility and reversibility of architectural features than others. Information architectures can reinforce societal biases and discriminations and lead both to prosocial outcomes and unintended social ills. Because information architectures are so tightly linked to economic activity, regulations that quell one social ill in the near term may have an inadvertent impact on social gains in the long term. A society may burn books to quell discord in the short run, only to discover that the information in those books was necessary to solve a future problem that has more enduring consequences.

Information architectures surely vary along many dimensions, and useful characterization of information architectures should allow us to highlight their differences. Here we highlight four dimensions along which we might characterize IAs, with the understanding that this list is not exhaustive. Indeed, future work should more precisely capture the “computational primitives” (cf.^32^) that allow elements of information architectures to create, redirect, organize, filter, curate, modify, or destroy information.

### Flows and structures

One way to characterize IAs is by their permissible and actual paths for information flow. Who is connected to whom? What are the possible paths for communication between individuals A and B? Flow entails directionality and often hierarchy. For example, broadcast media is characterized by one-to-many information flows, whereas peer-to-peer networks are many-to-many. This has changed as community opinion leaders transitioned from well-informed individuals who consume news to well-connected individuals who create engaging content on social media^4,5^. As with many complex systems, idealized formal models may help to characterize information architectures. Formal structures such as multiplex and multipartite networks may be useful in this regard, with nodes representing individuals, organizations, and other information sources and edges representing connections via various communication channels, including physical proximity^33^.

### Volume and bandwidth

How much information can be stored within various media and transmitted between individuals, and at what rate does that transmission occur? Many sociopolitical leaps in human cultural evolution have corresponded with changes to the volume and bandwidth capacities of those societies^34^, driven by a mixture of technology, infrastructure, and the institutionalization of teaching and pseudo-teaching (e.g., books, videos, and other media). In the past, information was severely limited by spatial proximity and by human physical and perceptual capacities, despite the importance of technological advances like the printing press^35^. Now, beyond the changes brought about by advances in transportation and physical infrastructure, the internet and digital technologies allow storage and rapid communication of nearly limitless amounts of information across nearly any physical distance. Sterelny^34^ invokes the evolutionary concept of niche construction to help explain some of these changes, as humans have actively adapted their informational environments to meet their communicative needs. It bears mentioning that the volume and bandwidth of IAs are also set by physical limits in the amount of energy required for an IA to gather, process, store, and distribute information. This is highlighted by current debates surrounding the ecological footprint of cryptocurrency and large language models^36,37^, both of which are likely to be significant influences on information flow but depend on massive increases in energy production.

### Distributional constraints

Not all information can be shared with the same ease. There are often legal and normative constraints over words, images, topics, and sentiments whose transmission is either encouraged or forbidden in certain contexts or by certain classes of people. In Germany, for example, imagery and sentiments associated with the Nazi regime are proscribed, and most direct advertisement of medical products is prohibited throughout the European Union. In the US, citizens can say nearly anything they want about politics but are restricted from making false claims about medical products, while non-citizens have limited rights regarding political speech. The Chinese government censors references to content, including the Tiananmen Square protests and massacre, Tibet and Hong Kong independence movements, state persecution of Uyghurs, and the resemblance between Chinese President Xi and Winnie the Pooh. Social media platforms may restrict or hide posts flagged as containing inflammatory content^38,39^. Logistical constraints can also influence distribution. For example, during the COVID pandemic, movies could still be released online while museums and the performing arts suffered from severely decreased patronage. Thus, IAs can be characterized by how different types of information content can be distributed between sets of nodes.

### Accessibility

Consider the idealized case in which all the information an individual could hypothetically access in society is represented as a massively long bitstring, with each bit representing a piece of information. An individual’s accessibility is the proportion of that bitstring that is available to them. This thought experiment allows us to consider how accessibility varies across society and to characterize inequality in access to information as a function of characteristics such as wealth, ethnicity, or social class. Accessibility may include access to archives or repositories, including libraries, websites, website APIs, code repositories, and digital memory banks—we might consider access to previously stored information as the *retrievability* of the IA. Accessibility can change over time for individuals as well as societies, both because new information is created and old information is destroyed, and also because infrastructures, laws, and norms may change.

These four dimensions allow us to compare contemporary or past societies in terms of their entrenched information architectures. In our toy examples, Harmonia is characterized by centralized and regulated information flows, limits to volume that allow for top-down monitoring, strict constraints to content distribution, and tiered information access based on social role. Libertaria is contrastingly characterized by a decentralized, peer-to-peer flow structure, with volume, distribution, and accessibility constrained only by practical concerns. Importantly, these examples are not meant to suggest that certain IA features are compatible only with authoritarian or democratic states. For example, a state-controlled authoritarian environment may co-opt an IA that has a network structure, bandwidth, and accessibility closer to that of a *Libertaria-*like society. It may adopt a “firehose of falsehood” strategy to “flood the zone” of a relatively open IA with misinformation to bury the truth in a flood of lies^40,41^. The intended result is widespread distrust of any information. Indeed, Hannah Arendt^42^ suggested that this type of dynamic may underpin the rise of totalitarianism from more democratic societies.

How and why do IAs with various characteristics come about? How do they shape human interaction, and how are they in turn shaped by those interactions? We turn to these questions in the next two sections. We will first use the characteristics described above to explore historical cases in which new information architectures led to wider downstream consequences. Then we will turn to elements of more contemporary information architectures, with a focus on internet applications.

## IA examples and illustrative case studies

In order to highlight the importance of understanding the dynamics of IAs, this section provides a series of historical comparisons within IAs at major moments of change and across IAs. We will look at shifts induced by the introduction of the first transatlantic cable in 1866 and by the introduction of cellular phone coverage along the coast of India from 1997 to 2001 and in Iraq from 2003 to 2008 during the civil war. For comparisons between contemporaneous societies with different IAs, we will look at differences in scientific progress between England and Spain in the 17th century.

The laying of the first successful transatlantic cable in 1866 marked a clear shift in the information architecture for the cotton trade. As discussed by Steinwender^43^, the cable reduced the time for information transmission from 7 to 15 days to just one day, providing more accurate and more timely forecasts of British and European market demands for cotton products originating in the United States. In terms of the four dimensions outlined in Section 3, the immediate effects were most obviously seen in changes to bandwidth, but the introduction of rapid transatlantic communication affected all four factors and created new affordances for further changes to come:

1. Flows and structures: The middlemen of shipping and postal carriers were shortcutted with the facilitation of more direct communication channels. Nevertheless, patterns of connectivity between principal actors in the cotton trade were not substantially altered.
2. Volume/bandwidth: There was a massive increase in the transmission rate for low data volume communications, e.g., short telegrams regarding prices or financial conditions. The ability to transmit large amounts of data (e.g., documents) was unchanged, as these still required physical movement from one place to another.
3. Distribution constraints: Industries with early access to cable transmissions gained an advantage in terms of meeting changing demand with appropriate supply, while the reduced economic costs of communication opened the door to greater competition from newer entrants to the market.
4. Accessibility: The transatlantic cable granted the connected actors (and nations) increased access to each others’ information, increasing the inequality in information access.

This new architecture had dramatic effects on economic flows and structures^43^. Price dispersion between New York and Liverpool markets dropped substantially—the average price difference fell by 35%, the equivalent of removing a 7% ad valorem tariff. Trade volumes also increased by 37% on average after the cable was introduced. The architecture enabled more efficient alignment of supply and demand across continents, with estimated efficiency gains of 8% of annual cotton export value from the US to Britain.

The information architecture enabled by undersea cables catalyzed major changes across multiple domains beyond economics. For example, they transformed business operations and logistics. News reporting also drastically changed, with outlets like Reuters even investing in cables to capitalize on access to faster international news^44^. More broadly, it impacted politics, culture, and society. The ability to almost instantaneously transmit messages revolutionized communications between governments, businesses, and individuals.

Over a century later, the introduction of cellular phone coverage around the world had similar impacts by enabling new kinds of immediate information flows. Here, we focus on two case studies of expanded cellular coverage in India and Iraq in the late 1990s and early 2000s. In terms of our four dimensions, cell phone coverage introduces a broader set of changes:

1. Flows and structures: Massive increase in interconnectivity.
2. Volume/bandwidth: Massive increase in rate for even high-bandwidth data communications.
3. Distribution constraints: Large increase in the ease of communication with anyone whose phone number is known, reducing reliance on face-to-face interaction.
4. Accessibility: Cellular customers could access information in more contexts and locations, while the costs of cellular service introduced inequalities in communication access.

Not surprisingly, the adoption of mobile phones catalyzed major shifts in market dynamics and economic outcomes. One study showed that when cellular coverage was introduced along the coast of Kerala, India, from 1997 to 2001, the price dispersion between local fish markets dropped substantially, with the coefficient of variation in prices falling from 60–70% to 15% or less almost immediately^45^. This enhanced integration enabled fishermen to eliminate the previous 5–8% of daily catch that was previously wasted due to saturation in local markets. Consumer prices for fish declined by 4% on average, while the improved efficiency increased fishermen’s profits by 8% on average, with those using phones gaining the most. Some fishermen reported compiling extensive contact lists with hundreds of potential buyers to survey prices before deciding where to sell their catch. Similar effects have been observed in other developing country agricultural markets^46^.

The ability to coordinate sales remotely revolutionized trading, transforming isolated village fish economies into integrated networks. Beyond the economic shifts, mobile connectivity impacted social relations and status. Fishermen with mobile phones rose in prominence, with their broader set of contacts making them valuable nodes for transmission of market information, even to those without phones. The examples of the telegraph cable and the adoption of mobile phones in Kerala demonstrate vividly how new architectures that enhance information flows can profoundly reshape economic and social landscapes.

In Iraq from 2003 to 2008, the gradual rollout of cellular coverage had more complex effects. There, insurgents used cell phones to “coordinate actions, execute attacks, and quickly react to counterinsurgency operations” and even threatened providers for not doing enough to maintain their networks^47^. From the perspective of Iraqi government forces and their U.S. allies, cellular coverage also made “it easier for the population to share information about insurgent activity, and to safely and anonymously call in tips” on insurgent activity^47^. The shifted IA created by cellular coverage offered select advantages to both sides, with fewer attacks overall and some evidence that insurgents shifted to using more indirect fire weapons, which could be used to attack from locations of the insurgent’s choosing.

Turning to comparisons across countries, it is instructive to compare England with Spain in the 1700s. If one had to guess in 1650 which country—Spain or England—would lead the flowering of science, choosing Spain would have been entirely reasonable. The country had a larger population, roughly 10 times as many universities, and a similar GDP per capita adjusting for purchasing power. It was England, however, rather than Spain, that established a nationally supported scientific society in 1660^48^, putting the nation’s imprimatur on the notion of open scientific discourse. In terms of our four dimensions, there were some clear differences between the countries, but also many similarities:

1. Flows and structures: Both nations had similar socioeconomic and technological constraints, though differences in culture and religion created somewhat different patterns of information flow.
2. Volume/bandwidth: Both nations had access to similar technologies and affordances for the storage and transmission of information.
3. Distribution constraints: Many ideas faced significant content-based restraints in Spain due to the ongoing influence of the Inquisition, while English culture promoted a freer flow of information through books, pamphlets, education, and public debate.
4. Accessibility: Spain had substantially more universities^49^ but lacked the government-funded scientific societies and social norms that made engagement in science a proud hobby for many wealthy individuals in England^50^. Curricula in Spanish universities were heavily scrutinized by the Inquisition.

From 1660 through 1700, Spain remained in the grip of the Inquisition. It banned the printing of any books viewed as countermanding Inquisition values, and preferentially targeted wealthy and highly educated individuals for persecution, reducing incentives to become educated, to publish controversial ideas, or to establish public institutions^51^. In England, the Royal Society was formed in 1660 by a royal charter from King Charles II, emphasizing the sort of mathematical and experimental inquiry described by Francis Bacon in his 1626 novel New Atlantis. Spain did not establish a national scientific society until 1790, as the Inquisition’s power was fading^52^. The subsequent difference in these countries’ contributions to science was massive. Seventeenth-century England saw a flourishing of research, with its scientists playing leading roles in many fields, while Spain experienced a century of stagnation and declining GDP, with the Inquisition leaving persistently lower levels of education and social trust in its wake^51^.

## Feedback between information architectures and individuals

Information architectures structure and influence the social activities of the actors embedded within them in at least two distinct ways. First, IAs enable social contact by providing channels through which information can flow. Second, IAs store information, granting actors powers of memory and recall beyond their otherwise immediate surroundings. When combined with IAs’ ability to scale communication, this often allows for cheap—albeit biased—information search. This, in turn, can influence the establishment and execution of social and political institutions, shaping peoples’ everyday cultural practices and evolving as the technologies, institutions, and practices that enable information architectures evolve.

Modern-day internet applications are important facilitators when it comes to the fulfillment of human needs to communicate, form relations, cooperate, and coordinate. For example, as of 2017, dating apps have become the primary means for heterosexual people in the U.S. to find romantic partners^53^. While some people do still find partners “the old fashioned way” (e.g., via shared activities or mutual friends), dating apps have taken over a central role in fulfilling the basic human need of finding a partner, likely because they reduce the costs of both search and communication^54^. Online dating lowers the cost of finding new contacts but also makes those contacts less likely to be deeply integrated into one’s regular social networks, thereby affording the cultural practice of “ghosting” (abruptly ceasing communication without explanation), which was relatively rare in the pre-app era^55,56^, when the reputational costs of ghosting were likely higher. The restructuring of information architectures has led to similar outcomes in other times and places. Among the Magar people of Nepal, for example, the introduction of mail and postage in their valley territory in the 1980s and 1990s restructured their system of arranged marriages, leading to the increase in elopements as a result of more efficient search among potential spouses^57^. Online, self-organized communities on Reddit are established through efficient information exchange^58^, providing support for topics and norms that would not typically be afforded in non-urban, geographically isolated communities in the pre-Internet era.

The shift of much human interaction from ephemeral face-to-face conversation to persistent computer-mediated discussion has brought about ubiquitous “data shadows”—vast archives of one’s comments, transactions, and other recordable behaviors. Much of this material is stored, owned, and potentially sold by the platforms on which the interaction took place. This has profound implications psychologically, socially, and legally as the past, instead of fading into a dim temporal distance, remains ever-present and easily searchable. Statements made for a particular situation and audience can resurface at any time, and in quite different contexts; this immediately changes people’s ability to keep information private and, eventually, transforms cultural conceptions of privacy and public space (Refs.^59,60^). Thus far, third parties have been the primary users of this information: governments pursuing criminals (or repressing dissent), advertisers targeting consumers; these uses are often harmful to the subject and creators of the information. Potentially, these archives could also be used by and for the benefit of their subjects and creators, e.g., for self-reflection, social navigation, or enriching online self-presentation^60,61^.

Because the information we send and receive shapes and constrains our mental lives and behavioral affordances, the components of information architectures shape how people build their cultural repertoires, networks, and shifting norms and values. Information architectures tend to amplify some information contents and dampen others; the information that is or is not transmitted can, therefore, reflect biases of the people who use it—or of the governmental or corporate organizations that control the information flows. This point was made by Herman and Chomsky^62^ in their analysis of the media—multiple stages of filtering produce media employees that promote the interests of the ruling classes without requiring any deliberate intent to do so on the part of those employees. More recent analyses have shown that continued increases in the rate of information transmission likely reinforce and amplify the biases produced by such filtering^63^.

Feedback can lead to co-evolutionary dynamics between IAs and their users. Because IAs determine how information is transmitted, cultural selection will tend to provide content best suited to the nature of the architectures, and that content may then provide further selection on the nature of the architectures^13,64,65^. While the medium may not exactly *be* the message^66^, the medium shapes the message, and the message shapes the medium. For example, the nature of a market shapes the products exchanged on it while also changing to accommodate new forms of exchange^67^.

The design of information architectures is rarely value-neutral^68^. As we have emphasized, IAs facilitate certain types of behaviors, norms, and ideas, while suppressing others. They are at least partly designed by stakeholders in accordance with their values, goals, and desired outcomes. The structure of information architecture is, therefore, the result of a dynamic process between the information architecture itself, those who utilize it, and those who contribute to its design (these last two need not be fully separable). This co-evolutionary dynamic creates at least three points for conflict: tensions between designers and users, between designers and IAs, and between IAs and users. Below, we walk through several recent examples of IA components (parts of cultural infrastructure that contribute to the overall IA) involving online information transfer. Although the following examples largely focus on IA components that were *deliberately* designed, it is important to stress that much of IA design results from non-deliberate processes that may be unconscious or emergent^31^.

Designers of IA components may come to find that their product is used in a manner inconsistent with their original intentions. Bitcoin, for example, was originally designed to serve as a competing currency with modern fiat currencies but is now largely utilized as an investment tool rather than as a form of liquid currency^69^. Tensions between designers and IA components may result when the structure of the system makes adaptation to fulfill a particular demand difficult (that is, meeting a critical goal is not among the set of what Kauffman^70^ has dubbed “adjacent possible” solutions). Such tensions arose in the relationship between Twitter and its designer Jack Dorsey, who spoke on Twitter’s entrenched design features in 2019, stating, “I don’t think I would even create ‘like’ in the first place because it doesn’t actually push what we believe now to be the most important thing, which is healthy contribution back to the network”^71^. Twitter is an especially interesting example, given the extreme changes to the social media platform following its acquisition by Elon Musk in 2022 and its subsequent rebranding as “X.” The rules and algorithms changed rapidly, followed by a dramatic decrease in the number of overall users^72^, an increase in hate speech propagated on the platform^73^, and a demographic shift rightward^74^.

Feedback between IAs, users, and designers can be cooperative as well as antagonistic. In some cases, features may be added that help the IA propagate information and similarly add features that users requested. On the other hand, unwanted changes can come about which are required by the platforms, but not by users. Two-factor authentication, for example, is often perceived by users as tedious but arises due to misuse of the architecture by a subset of the population. Finally, continued co-evolution can lead to stark changes in the value and affordances of some features. Consider, for example, the design of website “cookies,” which were originally designed to facilitate faster interactions between websites and their users. Over time, these were exploited by advertisers, who used cookies across websites to track user behavior. In response to this, EU legislation arose requiring users to actively grant consent on websites that used them^75^. More generally, co-evolution in under-regulated capitalist markets has occasionally (though perhaps increasingly often) led platforms that were initially optimized for user experience to increasingly subordinate that experience in favor of stockholder profits and corporate control, a process which Cory Doctorow^76^ has dubbed “enshittification”.

Another example is the feedback between social media companies, users, content algorithms, and “influencers”^5^. Social media companies create content algorithms to increase (or maximize) “user engagement”, which is defined by how much users use the platform. Content that increases engagement also increases revenue for the companies, which share their revenue with the users who generate the most engaging content: the “influencers”. Influencers are incentivized to create content that generates the most engagement because it results in more revenue for them. Influencers who do not continue to produce engaging content will lose influence and revenue. The content algorithms also typically promote content that is getting the most engagement, leading to a “rich-gets-richer” effect, with some influencers gaining huge followings relative to the typical user. This effect is observed even in academia: those who share their work on social media and have more followers are more likely to be cited^77^.

The speed at which information architectures are evolving appears increasingly to be much faster than the speed with which human societies can adaptively respond to them. IA components whose use was once optional can scale rapidly, becoming requirements for human integration into social networks. For example, in many industrialized nations, not having a smartphone creates numerous impediments, some of which have nothing to do with the technology’s traditional functions for person-to-person communication. While designers and users are both, at some level, active participants in information architectures and, therefore, both contribute feedback to the design of their components, users may feel helpless in terms of their ability to influence IA components or the societal dependence upon them. This is exacerbated when control over information architecture components is less democratic and egalitarian.

## The nature of the IA competition

Information architectures are not monolithic or uniform. As described above, different societies (or sectors of society) may be governed by different architectures. As such, IAs may be subject to intergroup competition, much as groups with different norms and institutions have competed throughout human history^31,78,79^. What do we mean when we say that information architectures compete? What do they compete over? What are the outcomes of the competition?

We find it useful to approach these questions from a neo-Darwinian perspective. IAs vary over aspects of their design—for example, over their structures, bandwidth, distributional constraints, and accessibility (see Section “What are information architectures?”). Some IAs are more likely to persist and expand, and others are more likely to wither or die. It is, therefore, tempting to conceptualize IAs as the products of variation and selective retention, resulting from well-understood processes of cultural evolution^80,81^. We take this approach here, though with caution. Information architectures are multifaceted cultural artifacts that require consideration of how collective traits evolve^31,82^. IAs also constitute the communicative niches of societies and, therefore, appear well-suited to be studied using the framework of “cultural niche construction”^83^, which considers the ways in which humans actively shape the selective pressures that consequently shape the evolution of our cultural variants. Rich theory on the evolution of collective phenomena like IAs is still underdeveloped; we hope that the discussion here helps progress in this area.

As with biological organisms, the evolutionary success of information architectures depends on the environments in which they compete. These environments include not just physical constraints, but the laws, rules, and norms that govern their use—what has been called the “up-code” by Shapiro (^84^; in contrast with “down-code”: the operating systems and network protocols that directly shape information flow). For example, in a state-controlled environment, successful IAs would have structural features and affordances that most closely align with the goals of the state^68,85^. As we described in Section “Characterizing information architectures”, a state-controlled authoritarian environment may, for example, favor one-to-many broadcast IAs where the state can distribute its message to the masses and control the flow of information. The state may also suppress many-to-many IAs, where any person’s messages can spread to any other, even those critical of the authoritarian regime. State control of broadcast media could also be combined with monitoring and suppression of many-to-many IAs, provoking the development of clandestine, though inefficient, IA components that evade government censors (such as VPNs).

In a more capitalist environment, successful IAs might be those that best attract, and especially monetize, flows from consumers for profit^4,85^. The two main models of this are subscription IAs, where individuals pay to receive and/or transmit information, and advertising IAs, where the information transmitted is incidental relative to the information that can be captured about users and sold to those who would pay for it. For subscription IAs, those that attract the most subscribers who are able and willing to spend the most money will tend to win. For advertising IAs, those that attract the most attention from the most people willing to spend money on advertised products will tend to win. This is not without its dangers. For example, the need to increase usage traffic may favor algorithmic IAs that attract and maintain attention by provoking outrage in users, which may, in turn, increase societal polarization when outrage is directed toward members of other social or political groups^86–88^. IAs implementing these have been successful competitors against other IAs, but arguably at an overall cost to societal well-being. Additionally, competition over which IAs can best collect and sell personal data will tend to favor IAs that feature the weakest privacy protections possible without provoking a backlash from users.

Because senders and receivers will tend to use the same IAs to transmit information, “networking effects” can create additional inefficient outcomes in IA competition. Networking effects, a form of lock-in, create social inefficiencies because it is difficult for a small number of users to switch to another IA unilaterally. The classic example of lock-in is the QWERTY keyboard, which was designed to be inefficient, reducing the number of sequential keystrokes close to one another to avoid jamming on mechanical typewriters. Because most people learn to type with a QWERTY keyboard and it is costly to manufacture and distribute alternate keyboard layouts, this inefficient layout persists well into the digital age. Similarly, large social media platforms are very difficult to unseat by more socially desirable alternatives, because this would require a critical mass of users to make the switch simultaneously. Proposals to legislate interoperability between platforms—making communication between users of different social media platforms as easy as between users of different telephone carriers—would change the consequences of these networking effects and allow smaller competitors to persist more easily^89,90^.

Environments less constrained by state control or capitalistic drive might favor IAs that produce public goods and are maintained by donations, volunteer labor, and institutions that favor collective action^91^. However, there is always the danger that such IAs will succumb to “enclosure” by corporations or states, which often have strong incentives to control information for their own ends^68,92^. It has been argued that the European Union has structured a regulatory environment based on human rights rather than either authoritarian or market forces and that this regulatory regime might ultimately become the primary environment in which IAs compete^93^. However, wide-reaching (e.g., multinational) IAs must compete—just as organisms do—by succeeding in *many* different regulatory environments that impose often contradictory constraints. For example, one can readily imagine how a “generalist” IA can facilitate communication across different sociopolitical environments by changing the rules and norms based on the environments involved in particular information transfer. Such an IA might eschew regulation and user privacy protections in a capitalist environment, accept regulation and user privacy in a rights-based environment, and help suppress speech and user privacy in an authoritarian environment.

So far, we have looked at competitions between IAs within specific environments, but as many IAs span the globe, they are increasingly becoming competitive domains between international actors, both state and non-state. Consider competition between states. State institutions set constraints on information architectures within those states. Some states assume control of the information within the state, some cede control largely to corporations. Some have strict privacy controls, others do not.

With the “information environment” becoming a prominent domain of international competition, and with states seeking greater influence over the information flows within other states^6^, it is difficult to predict which IAs will dominate. Will isolationist IAs in North Korea and authoritarian IAs in China maintain state power by limiting the flow of available information from the outside world and between citizens of those states? Will the relatively open IAs of Western countries continue to be susceptible to misinformation campaigns from abroad? Will states with privacy regimes that allow private firms to scoop up personal data be at a disadvantage when this data is sold to competing states for a small cost? When IA competition becomes a proxy for state competition, selection happens on “up-code” as well as “down-code”^84^.

What are the potential outcomes of IA competition? This will depend on physical and historical constraints as well as the selective pressures that lead users and designers to adopt or impose one system over another. One possible outcome is efficiency; IAs will become better at transmitting, compressing, or filtering information. For example, the printing press, the mail system, the telegram, the typewriter, the telephone network, radio, broadcast television, cable television, and satellite communications—all of these captured information flows from preceding technologies and allowed for more and more rapid and low-cost communication. However, this efficiency also often entails production or dissemination bottlenecks that lead to state or corporate capture, which can exacerbate authoritarianism, inequality, and other negative social outcomes. Outside of contravening forces, state-controlled IAs will become better tools of state control. IAs in capitalistic environments might get more efficient at attracting attention and advertising dollars by serving more and more polarizing content and selling more and more user information.

## Open questions and needed research on IAs

From the perspective of dual inheritance theory^94,95^, humans and IAs have been co-evolving for a long time. This makes sense given that IAs shape human communication, which in turn is entangled with our ability to cooperate and coordinate at scale, to learn socially, and to build (and treat as real) collective fictions like identity, stories, religion, currency, and laws that then act as causes of their own in the world^80,96,97^. From this perspective, information architectures are critical for enabling and constraining almost all of our truly social interactions and exchange, from how we coordinate to whom we learn from to which stories or fictions are disseminated and come to dominate populations and systems. Taken to a logical extreme, IAs are not just cultural and societal artifacts but are *intrinsic* components of cultures and societies, since without sufficient information transmission, incorporation, and storage, culture itself can begin to erode^98^.

Today’s IAs do not seem to be merely more of the same thing we’ve always had. That said, more of the same thing can be sufficient to provoke the emergence of a *new* thing. The increasing scale, reduced cost of information transmission, growing interconnection, and pervasive reach of modern IAs remind us that more *is* different. We need new research on IAs precisely because we cannot depend on historical case studies and precedent to help us understand—much less predict or prescribe for—today’s IAs. Their growing interconnectedness and dynamism mean we are living in a world shaped by IAs that reflect what Weaver^99^ prophetically called “organized complexity.” Far different from systems of “organized simplicity” (which can be tackled by studying a few variables) or “disorganized complexity” (which avail themselves to statistical methods), systems characterized by organized complexity, with its numerous variables “interrelated into an organic whole”, require new methods and approaches that include the use of mathematical models, computer simulations, and, perhaps most importantly, an interdisciplinary collaboration among experts from different fields^100^.

We are essentially trying to build a science to better understand IAs that are evolving at the speed of engineering—a speed that historically far outstrips the pace of science. This engineering is ushering in new IAs that are bringing humans and increasingly capable machines together to create new kinds of information storage, computation, and communication processes and capabilities^3^. Indeed, we may be witnessing the birth of new kinds of IAs. Further, IAs’ influence and interactions may plausibly begin to get even more complex, resulting in a host of consequences that our current scientific machinery is ill-equipped to predict or understand.

In addition to their scale, speed, and complexity, IAs also challenge existing scientific processes and tools because of some of their potentially unique features that make research on IAs different—and arguably harder—than research on other socio-technical phenomena. IAs can be difficult to cleanly delineate compared to more discrete systems, and their scope, interaction, and even co-dependence can be fuzzy. IAs also evolve dynamically in response to technology and human behavior, leading to nonlinear interactions among IAs’ up-code and down-code. Such recursivity also means it is not clear whether—to borrow from antiquity—we can ever step into the same IA twice. All of this is confounded further by the opacity of many IAs; they lack the transparency researchers ideally look for (though transparency in socio-technical systems can be a double-edged sword;^101^). We hope to better understand IAs’ normative nature: like many sociotechnical systems, IAs necessarily encode (even if only implicitly) and promote specific value systems. That is, IAs shape information creation, dissemination, and use at levels ranging from underlying infrastructure to daily user experience. Integrating research across these levels to understand the bigger picture and to guide interventions poses non-trivial technical, methodological, and even ethical challenges.

Considering how IAs present both old and new research problems, understanding them can seem overwhelming. But if trends hold, we need to significantly increase the speed, diversity, and value of research on IAs to increase the chances that we better understand them and can shape their design to benefit democratic societies. This will require creativity and adaptability to navigate their complexity, dynamics, opacity, and entanglement with human behaviors and values. Developing a systematic understanding and tools to meaningfully improve IAs in ethical ways will require confronting these unique properties head-on.

To help structure future IA research in a way that we hope will be useful and intelligible, we propose a three-pronged analytic framework adapted from Naugle et al.^102^: *Predicting, Understanding, and Prescribing (PUP)* interventions for complex sociotechnical systems like Information Architectures. We believe this framework can help categorize and advance research on different questions related to IAs while helping to organize results in a systematic way that advances our collective knowledge. Further, the framework also helps underscore the need for creative experimentation and novel tools that will help us make progress across all three of those areas.

### *Predicting* the evolution and impacts of Information Architectures

New advances in computational power, data availability, analytic techniques, and theory promise to make prediction more tractable for IAs^103,104^. With a focus on creating predictive models, there may be new research approaches that allow us to better anticipate things like the trajectories of, the emergence of behaviors within, and social impacts stemming from specific IAs. With these approaches, we could better identify key parameters and variables that are the hallmark of persistent and adaptive IAs, and better forecast the outcomes of IA competitions. Without ignoring the difficulty of prediction in the face of organized complexity—a difficulty that should not be underestimated—being able to better assess futures as possible, plausible, or probable could better help us to anticipate how IA competitions lead to a range of potential outcomes: dominance of one IA over others, subordination of an IA to others, changes in assortment or polarization in or across IAs, the emergence of hybrid or syncretic IAs, and the impacts of IAs on the kinds and quality of information shaping a population or populations.

Some key research questions we have identified related to prediction and IAs are:

- What new research approaches can be developed to predict the social impacts and emergent behaviors of specific IAs?
- How can we identify the key variables that determine the persistence and “fitness” of IAs?
- How can predictive models be utilized to forecast the outcomes of IA competitions?
- What are the practical or theoretical limits to prediction in the context of IAs?
- How can we categorize futures as possible, plausible, or probable for IA competitions and their diverse outcomes?
- What methods and approaches are needed to model IAs with sufficient complexity and fidelity?
- How can we create effective ways to test predictions and validate models in IA research?
- What strategies can be used to rapidly learn from incorrect predictions in IA research?

Tackling these kinds of research questions is likely to require significant advances in methods and approaches so researchers can not only model IAs and their interactions with sufficient complexity^105^ but create new ways to test predictions that help validate and refine those models. In particular, research here demands creative thinking about how to gather feedback on predictions that can rapidly enable us to learn where and when we are wrong. Recent advances in using LLMs to simulate social networks of discursively coherent agents offer a promising direction^106–108^, though caution should still be exercised before drawing conclusions from AI models that are ultimately based on reproducing patterns in published corpora^109^.

### *Understanding* information architectures

Understanding might seem to be an obvious priority for outlining a research agenda for IAs, but the organized complexity of IAs presents significantly different challenges than predicting their characteristics and dynamics. There is work needed to understand their causal mechanisms, their characteristic features, and their informational boundaries. Prediction entails getting the right answers, understanding entails asking the right questions.

To better understand what IAs are and how they work, we need to tackle key research questions that involve theoretical and empirical research, including:

- How do we identify/draw boundaries around Information Architecture? What defines the scope and limits of an IA?
- What drives competition among IAs? What are the core fitness functions or units of selection for IAs?
- Are there inherent chaotic dynamics within IAs that could restrict our ability to understand and influence IAs?
- Are there fundamental limits to our ability to fully know an IA’s structure and boundaries? Are there impossible IAs that we can exclude on principle?
- When does something qualify as an IA? Is the demarcation primarily structural or functional? Are there useful distinctions between “true” IAs and other related structures in terms of real-world impacts?
- What is the relationship between IAs and the actors that create/propagate them? To what extent are IAs constrained by their core media (smartphones, SMS, DMs, etc.)?

Understanding IAs will require a significantly funded and coordinated research campaign if we are to build a mature science in this space: a community of researchers, a common infrastructure with standardized terms and measures, a body of confirmatory research and validated theories, and an engagement with well-intentioned policymakers to apply this understanding.

### *Prescribing* interventions for information architectures

Discussing applications of understanding leads to the third need for IA research focused on prescription: that is, designing interventions to create mechanisms for coordination and establishing collectively desirable outcomes. IAs pose distinct challenges here because of their influence on key human characteristics like health, wealth, and security, not to mention emotion, morality, and social identity. This means that prescription research will also need to navigate these challenges within and across different societal norms and regulations, especially as those norms and regulations are likely to vary across political and cultural contexts. Interventions in complex social systems are fraught with peril due to the inevitability of unintended consequences, but lack of action may be unacceptable if the alternative outcomes are worse. While we anticipate some discomfort around the topic of prescribing interventions for IAs (and feel some of that discomfort ourselves), IAs are already being intervened upon, so understanding the consequences of those interventions seems prudent.

Some of the key questions we need to answer to effectively influence, engineer, design, and control IAs for collectively beneficial outcomes include:

- What creative coordination mechanisms can we use beyond top-down government regulation? How can we harness collective intelligence and market dynamics? What other design mechanisms can be explored?
- What are the rationales for proposed interventions succeeding in shaping IAs? What would we measure to determine the impact or direction of the effects of interventions?
- How can we establish collectively desirable IA outcomes? What are the critical tradeoffs among participants involved in these outcomes?
- How can we extrapolate from historical examples of IA transitions and interventions to prescribe future actions? How can we use comparative analysis of IA competition and evolutionary scenarios to identify key intervention points?
- How can we map, manage, and/or design incentives that reflect the complexity of IAs and their constituent parts (which will increasingly also include capable machines, as agents, that respond to rewards, policies, and utility functions)?

As with the other research foci, addressing these questions will require creative approaches that help navigate the practical, theoretical, and ethical challenges that each question presents individually as well as collectively. Tools that can improve counterfactual reasoning, which is necessary to pose and answer “what if?” questions, include simulations for predicting how IAs will work under different scenarios, as well as causal inference and mechanistic models. The irony is not lost on us that we will also almost certainly need the very computational capabilities that are now increasing the complexity of IAs in order to better predict, understand, and prescribe for them going forward.

## Conclusions

As our social and information systems become more deeply entwined, the study of information architectures will be a tool for understanding and potentially shaping the techno-social environment that influences our lives. This paper makes an attempt to define, illustrate, and characterize the key dimensions of IAs. By delving into the complexity, opacity, dynamism, and co-evolution of IAs with human behavior, we can avoid the perception of being a victim of technological change. Instead, we can gain insights into the underlying forces driving transformation and identify opportunities for human agency in shaping the development of and interaction between IAs. The scope of this research will require innovative approaches, interdisciplinary collaboration, and a recognition of its significant challenges. We covered vignettes and historical examples that offer some simple clues to how to understand and research IAs and proposed a research framework focused on three key areas: prediction, understanding, and prescription. This framework was chosen to emphasize the proactive nature of the research agenda. By predicting the evolution and impacts of IAs, understanding their intricate workings and boundaries, and prescribing interventions for coordination and beneficial outcomes, we can perhaps steer society to adopt and evolve IAs that align with our collective values and aspirations. It is essential that we study not only individual online services and platforms but also frame the larger problem of IAs as a critical research domain. The influence of IAs extends beyond any single application, shaping our economies, politics, cultures, and social interactions in profound ways.

Navigating ethical challenges and diverse societal norms involved in shaping IAs will require ongoing dialog, experimentation, and adaptation. By working together across disciplines and sectors, we can strive to create IAs that promote human flourishing and collective well-being. The study of information architectures represents a critical step in this journey, empowering us to understand and shape the techno-social fabric that increasingly defines our world.
